# Are some breast cancers sexually transmitted?

**DOI:** 10.1038/sj.bjc.6603502

**Published:** 2006-11-28

**Authors:** J S Lawson, C-Y Kan, B J Iacopetta, N J Whitaker

**Affiliations:** 1 School of Biotechnology and Biomolecular Sciences, University of New South Wales, 36 The Point Road, Woolwich, NSW 2110, Australia

**Sir**,

High-risk human papilloma viruses (HPVs) have been consistently identified in 13–86% of breast tumours in the 10 studies published since 1999 (Lawson *et al*, 2006). High-risk HPVs of the same type have been identified in both cervical and breast cancer that had occurred in the same women (Hennig *et al*, 1999; Widschwendter *et al*, 2004). This observation has lead to the hypothesis that HPVs may be transmitted to the breast during sexual activities (Kan *et al*, 2005). If this hypothesis is correct, it is likely that HPV-positive breast cancers would occur in women younger than those with HPV-negative breast cancer. This is because HPV genital infections are much more common in young women who have had multiple sexual partners (IARC, 1995).

There are only two studies in which the age of women with HPV-positive and -negative breast cancer has been published. There were no differences in the average of age of women with either HPV-positive and -negative breast cancer in a study of Brazilian women (Damin *et al*, 2004). This is in contrast to a recent study of Greek women in which those with HPV-positive breast cancer were of average age 38 years as compared to average age 53 years for women with HPV-negative breast cancer (*P*-values for difference=0.001) (Kroupis *et al*, 2006).

We have reviewed the ages of Australian women with HPV-positive and -negative breast cancer in our study published in this Journal (Kan *et al*, 2005). These data are shown in Table 1. The average age of women with HPV-positive breast cancer was 55.6 years as compared to 63.8 years for women with HPV-negative breast cancer (*P*-values for difference=0.049). These data are compatible with the hypothesis that HPV-positive breast cancers occur in younger women than those with HPV-negative breast cancers, and that high-risk HPVs may have been transmitted by sexual activity with HPV-positive sexual partners.

## Figures and Tables

**Table 1:** Age of Australian women with HPV-positive and -negative breast cancer

	**HPV positive**	**HPV negative**
Number of women	24	26
Average age (years)	55.6	63.8

HPV=human papilloma virus.

*P*-value for difference in average ages=0.049, which is significant at the 95% level.
